# circHUWE1 Exerts an Oncogenic Role in Inducing DDP-Resistant NSCLC Progression Depending on the Regulation of miR-34a-5p/TNFAIP8

**DOI:** 10.1155/2021/3997045

**Published:** 2021-12-03

**Authors:** Xueliang Yang, Quan Sun, Yongming Song, Wenli Li

**Affiliations:** No.1 Department of Thoracic Surgery, Shanxi Provincial Cancer Hospital, Taiyuan, Shanxi, China

## Abstract

**Background:**

Circular RNAs (circRNAs) are reported as competing endogenous RNAs (ceRNAs) and play key roles in non-small-cell lung cancer (NSCLC) progression. Thus, this study was aimed at clarifying underlying molecular mechanisms of circHUWE1 in NSCLC.

**Methods:**

The quantitative real-time polymerase chain reaction (RT-qPCR) and western blot analyses were used for examining circHUWE1, microRNA-34a-5p (miR-34a-5p), and tumor necrosis factor alpha-induced protein 8 (TNFAIP8). IC_50_ of cisplatin (DDP) in A549/DDP and H1299/DDP cells and cell viability were analyzed by the Cell Counting Kit 8 (CCK-8) assay. Colony forming assay was performed to assess colony forming ability. Cell apoptosis and cell cycle distribution were determined by flow cytometry. Migrated and invaded cell numbers were examined by transwell assay. The association among circHUWE1, miR-34a-5p, and TNFAIP8 was analyzed by dual-luciferase reporter and RNA immunoprecipitation assays. A xenograft experiment was applied to clarify the functional role of circHUWE1 *in vivo*.

**Results:**

circHUWE1 was upregulated in NSCLC tissues and cells, especially in DDP-resistant groups. circHUWE1 downregulation inhibited DDP resistance, proliferation, migration, and invasion while it induced apoptosis and cell cycle arrest of DDP-resistant NSCLC cells, which was overturned by silencing of miR-34a-5p. TNFAIP8 was a functional gene of miR-34a-5p, and the suppressive effects of miR-34a-5p overexpression on DDP-resistant NSCLC progression were dependent on the suppression of TNFAIP8. circHUWE1 inhibition also delayed tumor growth of DDP-resistant NSCLC cells.

**Conclusion:**

circHUWE1 functioned as a promoter in DDP-resistant NSCLC by interaction with miR-34a-5p-TNFAIP8 networks, providing novel insight into DDP-resistant NSCLC diagnosis and treatment.

## 1. Introduction

Non-small-cell lung cancer (NSCLC) is a most common pulmonary subtype and is a major reason of tumor-caused death all over the world [1, 2], accounting 80% proportion of primary lung cancer [3]. Although a considerable amount of research has been done to investigate lung cancer treatment, the mortality rate of NSCLC patients is increasing [4]. Except for surgery, chemotherapy, targeted therapy, and immunotherapy have been developed for cancer pharmacological treatment [5, 6]; furthermore, novel promising anticancer approaches including cell therapy have been evolved in preclinical studies [7]. However, innate or acquired pharmacological resistance represents important obstacles to overcome. As well-documented, noncoding RNAs including circular RNAs (circRNAs) and microRNAs (miRNAs) are one of the pharmacological resistance mechanisms in tumor cells [8, 9]. Cisplatin (DDP) is the backbone of first-line chemotherapy for NSCLC [10]. Therefore, exploring the pathogenesis mechanism of DDP resistance is important for NSCLC physiotherapy.

circRNAs are the endogenous transcripts with a covalently closed continuous loops that form via linking the end of a 3′ exon with an upstream 5′ exon [11, 12]. Indeed, recent evidence supported that circRNAs acted as a critical regulator in the development of tumorigenesis [13]. For instance, circ_0000003 could regulate NSCLC cell malignant behaviors through mediating miR-338-3p [14]. circHUWE1 (hsa_circ_0004396) is coded by HUWE1 gene and located on chrX (53641494-53641706). A previous microarray profiles suggested a series of highly expressed circRNAs, including circHUWE1 [15]. We hypothesized that circHUWE1 played a carcinogenic role in NSCLC tumorigenesis.

miRNAs, single strand and noncoding RNAs, could modulate almost all biological processes [16]. Mechanically, miRNAs could target the 3′ untranslated region (3′UTR) of target mRNAs, thereby participating regulation of corresponding mRNAs expression [17]. miR-34a-5p dysfunction has been revealed in different cancers, and miR-34a-5p could be targeted by circRNAs to act as a tumor suppressor role [18–20]. Nevertheless, little is known on the relevance of circHUWE1 and miR-34a-5p in NSCLC.

Tumor necrosis factor alpha-induced protein 8 (TNFAIP8) is a member of a founding member of the TIPE family [21]. The upregulation of TNFAIP8 was found in a variety of tumors, which was highly linked to antiapoptotic and oncogenic gene expression [22, 23]. In addition, TNFAIP8 also played a significant role in drug resistance of tumor cells [24]. Therefore, the underlying regulatory function of TNFAIP8 involved NSCLC was investigated.

Currently, we analyzed circHUWE1, miR-34a-5p, and TNFAIP8 abundances in NSCLC tissues and cells and investigated their biological effects by functional experiments. We identified that the circHUWE1/miR-34a-5p/TNFAIP8 axis was associated with the progression of NSCLC, providing a new regulatory mechanism in NSCLC.

## 2. Materials and Methods

### 2.1. Patient's Tissues

NSCLC tissues were obtained from NSCLC patients (*n* = 56) at Shanxi Provincial Cancer Hospital, and adjacent nontumorous tissues were used as controls (*n* = 56). The written informed consent was provided by all participants. All removed tissues were frozen at -80°C. NSCLC patients were divided into two groups based on the patient's response to DDP, including sensitive group (*n* = 20) and resistant group (*n* = 36). This research was permitted and supervised by the Ethics Committee of Shanxi Provincial Cancer Hospital.

### 2.2. Cell Lines

NSCLC cells (549 and H1299), cisplatin resistant cells (A549/DDP and H1299/DDP), and control lung epithelial cells (BEAS-2B) were acquired from Nanjing Key Gen Biotech (Nanjing, China). Cells were cultured in RPMI 1640 medium (Biochrom KG, Berlin, Germany) containing 10% fetal bovine serum (Invitrogen, Carlsbad, CA, USA) at 37°C with 5% CO_2_. To mimic cisplatin resistance, A549/DDP and H1299/DDP were cultured with DDP (Invitrogen) with a final concentration of 1 *μ*g/mL.

### 2.3. Quantitative Real-Time Polymerase Chain Reaction (RT-qPCR)

TriQuick Reagent (Solarbio, Beijing, China) was used to extract total RNA in accordance with the manufacturer's protocols. Total RNA was used as a template converted into cDNA by Reverse Transcriptase Kit (CapitalBio, Beijing, China) or microRNA Reverse Transcription Kit (CapitalBio). After that, the cDNA was diluted and used for RT-qPCR using SYBR® qPCR Mix (Invitrogen) under the Roche LC480 system (Roche Applied Science, Mannheim, Germany). The changes of RNA were determined by a comparative threshold cycle (Ct) method, with glyceraldehyde 3-phosphate dehydrogenase (GAPDH) or U6 as controls. The primers were as follows: circHUWE1 (upstream—5′-GAAGTACAGGCCATGCAGAGCT-3′, downstream—5′-GATGGCTTCTGACAGATGT-3′); HUWE1 (upstream—5′-TGCCAGTGCTTGTAAGGAACT-3′, downstream—5′-TGGTGACAAATGTTATCTGGTCC-3′); miR-34a-5p (upstream—5′-GCCGAGTGGCAGTCTTAGCT-3′, downstream—5′-CAGTGCAGGGTCCGAGGTAT-3′); TNFAIP8 (upstream—5′-GATTCTGAGCAAAATAGCCAGCA-3′, downstream—5′-GGCTTCCTTCTTGTTGTGTGT-3′); U6 (upstream—5′-ATCCTTACGCACCCAGTCCA-3′, downstream—5′-GAACGCTTCACGAATTTGC-3′); and GAPDH (upstream—5′-TGAACCATGAGAAGTATGAC-3′, downstream—5′-TCTTACTCCTTGGAGGCCA-3′). Furthermore, isolation of RNA from NSCLC cells cytoplasmic and nuclear fractions was done with PARIS™ Kit (Ambion, Foster City, CA, USA).

### 2.4. RNase R and Actinomycin D Treatment

For RNase experiments, 10 *μ*g of total RNA was treated with 3 U/mg RNase R (Invitrogen) to degrade linear RNAs for 30 min at 37°C. In addition, 2 mg/mL of actinomycin D was purchased from Sigma-Aldrich (Merck KGaA, Darmstadt, Germany) and then was used to assess the stability of RNAs.

### 2.5. Transfection Assay

The short hairpin RNA against circHUWE1 (sh-circHUWE1) and shRNA control (sh-NC), TNFAIP8-upregulation vector (TNFAIP8), and control group (pcDNA) were designed by HanBio (Shanghai, China). The mimics of miR-34a-5p and control (miR-34a-5p and miR-NC), inhibitors of miR-34a-5p, and control (anti-miR-34a-5p and anti-miR-NC) were acquired from Genomeditech (Shanghai, China). 50 nM of miRNA mimic, 80 nM of miRNA inhibitor, or 0.5 *μ*g of plasmid vector was transfected into NSCLC cells by Lipofectamine 3000 (Thermo Fisher Scientific, Carlsbad, CA, USA) following the user's guideline.

### 2.6. Cell Proliferation

A total of 3000 NSCLC cells were added to each well of 96-well plates with 0.2 mL of culture media and then cultured at 37°C in a humidified atmosphere with 5% CO_2_. After that, cells were interacted with Cell Counting Kit 8 (10 *μ*L of CCK-8; Invitrogen) for 2 h. The optical density was assessed on a multiwell scanning spectrophotometer (BioTek, Winooski, VT, USA). IC_50_ values of the DDP treatment were determined using SPSS software (version 19.0, IBM, Chicago, IL, USA). Moreover, colony forming was conducted as previous description [25].

### 2.7. Flow Cytometry Assay

The single-cell suspension of NSCLC cells was prepared and fixed by 75% ethanol at 4°C for 12 h. The binding buffer containing Annexin V-fluorescein isothiocyanate and propidium iodide (BD Biosciences, San Diego, CA, USA) was used to stain cells in the dark at room temperature for 15 min, following by apoptosis assay under the flow cytometry (Becton Dickinson, San Jose, CA, USA). For cell cycle measurement, transfected NSCLC cells or control was collected and then fixed by 75% ethanol at 4°C for 12 h. After that, cells were incubated with 1 mL of PI/TritonX-100 staining solution containing RNase A for 30 min. Flow cytometry (Becton Dickinson) was used examine cell cycle, and ModFit software (Becton Dickinson) was used for data analysis.

### 2.8. Transwell Assay

The migration assay was performed via 24-well transwell chamber (Millipore, Billerica, MA, USA). In brief, cell suspension (3 × 10^5^ cells/mL) was produced using serum-free medium and then introduced into the upper compartment, while complete medium was used as nutrient in the lower chamber. The migratory cells were fixed by 75% ethanol at 4°C for 12 h and then stain by 0.2% crystal violet (BestBio, Shanghai, China). Cell numbers were determined on an inverted microscope (Mshot, Guangdong, China) at 100x amplification. 24-well transwell chamber with coated with Matrigel (Millipore) was used for invasion assay.

### 2.9. Dual-Luciferase Reporter Assay

Three bioinformatics prediction platforms, were utilized for prediction the binding miRNAs of circHUWE1, including Circinteractome (https://circinteractome.nia.nih), Starbase (http://starbase.sysu.edu.cn/), and circBank (http://www.circbank.cn/). Starbase was also used to predict complementary sequences between miR-34a-5p and TNFAIP8. The segments of circHUWE1 or TNFAIP8 3′UTR harboring miR-34a-5p-matched interacting sites or mutations of miRNA-binding sites were individually synthesized and inserted into pGL3-basic vectors (Realgene, Nanjing, China). NSCLC cells were cotransfected with 50 ng of transfected pGL3-basic vectors with 30 nM of miR-34a-5p mimic or control by Lipofectamine 3000 (Thermo Fisher Scientific). Dual-Luciferase Reporter Assay Kit (Thermo Fisher Scientific) was used for measuring luciferase activities.

### 2.10. RNA Immunoprecipitation (RIP) Assay

Approximately, 1 × 10^7^ cells NSCLC cells were resuspended in RIP lysis buffer from Imprint® RNA immunoprecipitation kit (Sigma-Aldrich) at 4°C. Subsequently, the cell extract was interacted with magnetic beads embracing IgG or AGO2 antibody at 4°C for 4 h. After purifying by proteinase K treatment, then isolated immunoprecipitated RNA was subjected to RT-qPCR.

### 2.11. Western Blot Assay

NSCLC tissues or cells were lysed by ice-cold lysis buffer (Cell Signaling Technology, Danvers, MA, USA). After quantitation, the extracted protein was loaded onto 10% SDS-polyacrylamide gel electrophoresis and then electroblotted onto clear blot membranes (ATTO, Tokyo, Japan). After blocking by 5% skim milk, the transferred membranes were probed with antibodies at 4°C for 4 h, including anti-TNFAIP8 (ab195810; 1 : 1500 dilution; Abcam, Cambridge, MA, USA), anti-Proliferating Cell Nuclear Antigen (PCNA; ab92552; 1 : 1500 dilution; Abcam), anti-matrix metalloproteinases 13 (MMP 13; ab51072; 1 : 1500 dilution; Abcam), anti-Cleaved Caspase-3 (c-caspase 3; ab32351; 1 : 1500 dilution; Abcam), and anti-*β*-actin (ab8227; 1 : 2000 dilution; Abcam) was served as a loading reference. After incubating with horseradish peroxidase-conjugated secondary antibodies (ab1500771; 1 : 3000 dilution; Abcam), the blots were visualized by enhanced chemiluminescence detection kit (GE Healthcare, Piscataway, NJ, USA).

### 2.12. *In Vivo* Experiment

Animal experiment was authorized by the Institutional Animal Care and Use Committee of Shanxi Provincial Cancer Hospital and performed based on the guidelines of the China Science Review of Laboratory Animal Welfare (GB/T 35892-2018). The BALB/c nude mice were obtained from Vital River Laboratory (Beijing, China) and then assigned to four groups (6 mice per group). The A549/DDP cells (2 × 10^7^) transfected with sh-circHUWE1 or control were implanted into the right back near the forelimb of BALB/c nude mice. Furthermore, BALB/c nude mice in the DDP groups were treated with 5 mg/kg of DDP every week by intraperitoneal injection after 7 d injection, with phosphate-buffered saline as control. The volume of xenograft tumor was calculated according to the equation: volume = 1/2 (length × width^2^). The xenograft was resected for weight measurement and then stored for further research. For immunochemistry for Ki-67 and TNFAIP8, a study was conducted as previous description [26] using Ki-67 (ab231172; 1 : 100 dilution; Abcam) and TNFAIP8 immunochemistry kit (#Yaji Biological, Shanghai, China).

### 2.13. Statistical Analysis

RNA or protein expressed values of each sample were assessed by SPSS software and presented as the mean ± standard deviation. The differential significance was performed by Student's *t*-test or analysis of variance followed by Bonferroni's test, and *P* value less than 0.05 was considered statistically significant. Pearson's analysis was used to analyze the correlations.

## 3. Results

### 3.1. Upregulation of circHUWE1 Was Related to DDP Resistance

**Figure 1:**
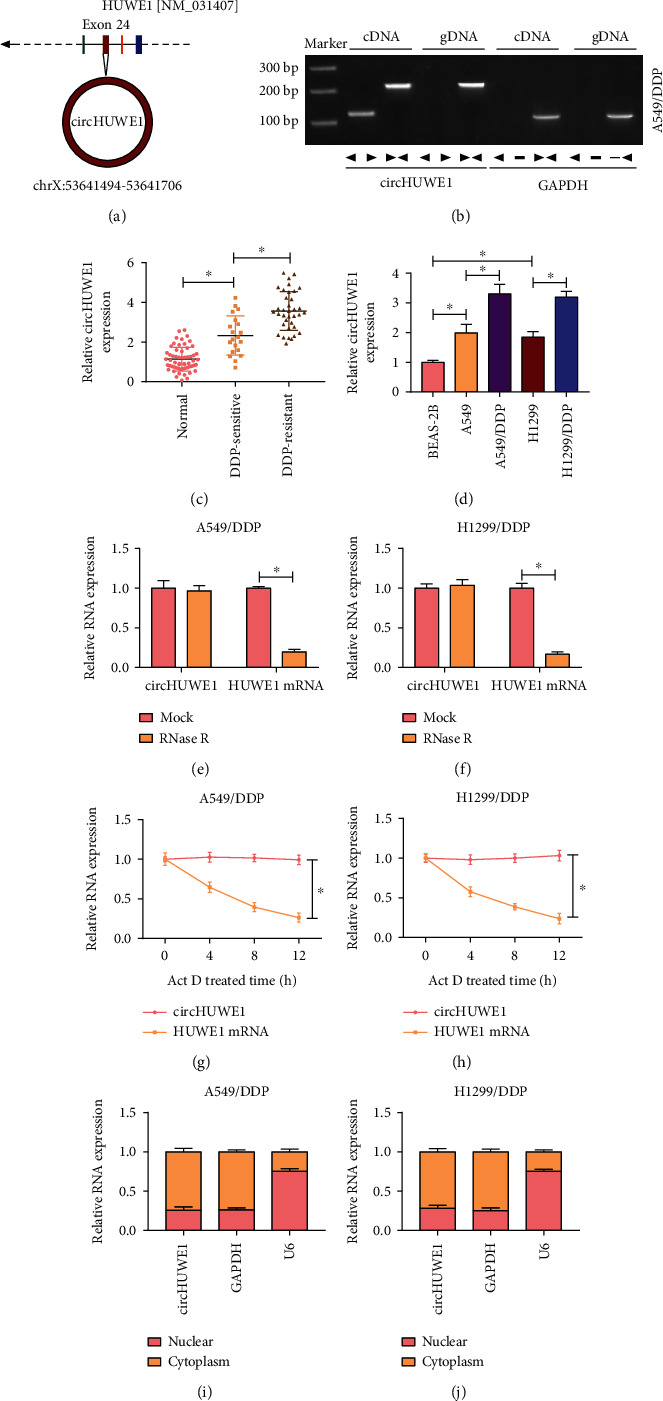
The expression level of circHUWE1 in NSCLC tissues and cells. (a) circHUWE1 was derived from the HUWE1 exon24. (b) PCR detected circHUWE1 and GAPDH using divergent primers and convergent primers in A549/DDP cells. (c) The relative expression of circHUWE1 was analyzed by RT-qPCR in adjacent non-tumorous tissues (*n* = 56), DDP-sensitive tissues (*n* = 20), and DDP-resistant tissues (*n* = 36). (d) RT-qPCR was used to assess circHUWE1 level in NSCLC cells and control BEAS-2B cells. (e–h) After treating with RNase R or actinomycin D, the relative RNA levels of circHUWE1 and HUWE1 mRNA were determined by RT-qPCR. (i, j) Cytoplasmic and nuclear fraction RNAs were isolated for measuring circHUWE1 level in cytoplasm and nuclei. ^∗^*P* < 0.05.

In this study, we will investigate the role of circHUWE1 in NSCLC. As shown in Figure 1(a), circHUWE1 was a transcript of the HUWE1 gene exon 24. Convergent and divergent primer amplification showed that convergent primers amplified circHUWE1 products from both cDNA and gDNA, while divergent primers amplified circHUWE1 from cDNA only (Figure 1(b)). We also found that circHUWE1 was upregulated in NSCLC tissues and cells relative to controls; importantly, circHUWE1 level was higher in DDP-resistant tissues and cells than DDP-sensitive tissues and cells, suggesting that circHUWE1 was related to DDP resistance (Figures 1(c) and 1(d)). Additionally, RT-qPCR revealed that circHUWE1 can resist RNase R degradation (Figures 1(e) and 1(f)). We also noticed that the half-life of circHUWE1 was longer than the half-life of HUWE1 mRNA, and circHUWE1 was mainly expressed in the cytoplasm of A549/DDP and H1299/DDP cells but not the nucleus (Figures 1(g)–1(j)). The association of circHUWE1 and DDP resistance was investigated in NSCLC.

### 3.2. Depletion of circHUWE1 Could Suppress the Progression of DDP-Resistant NSCLC

**Figure 2:**
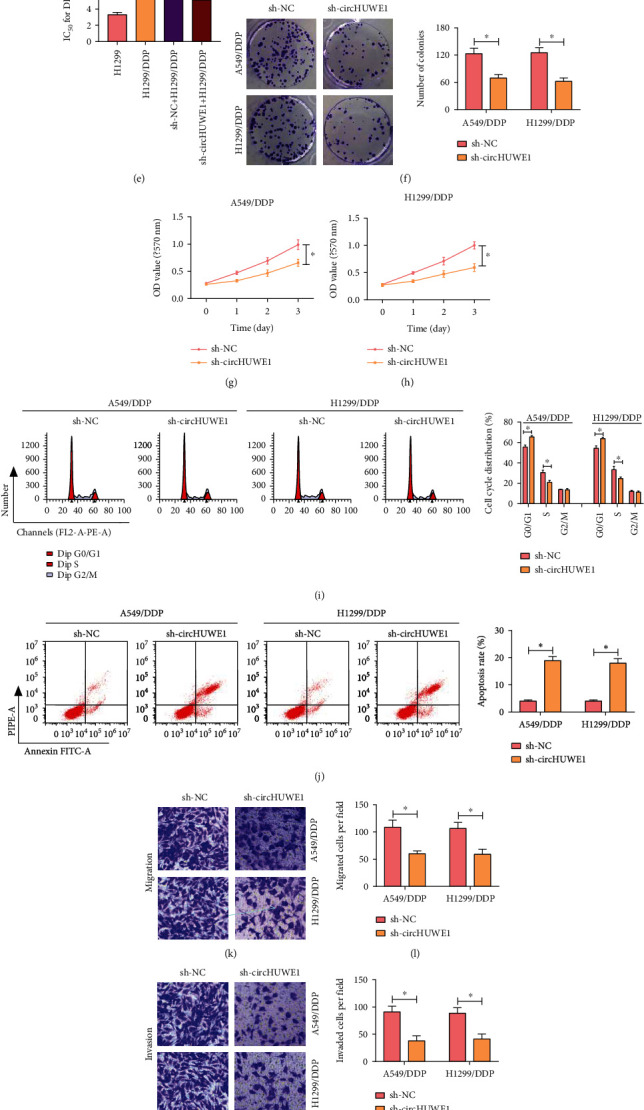
Effects of circHUWE1 downregulation on DDP resistance, proliferation, apoptosis, cell cycle, migration, and invasion of NSCLC cells. (a–q) A549/DDP and H1299/DDP cells were transfected with sh-circHUWE1 or sh-NC. (a) The expression of circHUWE1 was assessed by RT-qPCR. (b–e) IC_50_ of DDP in NSCLC cells was analyzed by the CCK-8 assay. (f) Colony forming assay was performed in A549/DDP and H1299/DDP cells. (g, h) The proliferation ability of A549/DDP and H1299/DDP cells was measured by the CCK-8 assay. (i, j) Cell apoptosis and cell cycle distribution were determined by flow cytometry. (k–n) Migrated and invaded cell number were analyzed by transwell assay. (o–q) The protein levels of PCNA, c-caspase 3, and MMP 13 were assessed by western blot assay. ^∗^*P* < 0.05.

To systematically illustrate the role which circHUWE1 played in NSCLC, A549/DDP and H1299/DDP cells were transfected with sh-circHUWE1. circHUWE1 was greatly decreased in A549/DDP and H1299/DDP cells after transfection with sh-circHUWE1 (Figure 2(a)). Moreover, the elevations of IC_50_ in A549/DDP and H1299/DDP cells compared with A549 and H1299 cells were blocked by circHUWE1 inhibition (Figures 2(b)–2(e)). The results of colony forming and CCK-8 assays suggested that depletion of circHUWE1 could inhibit proliferation ability of A549/DDP and H1299/DDP cells (Figures 2(f)–2(h)). The silencing of circHUWE1 also induced cell cycle progress arrest at S phase and cell apoptosis in A549/DDP and H1299/DDP cells, as confirmed by flow cytometry assay (Figures 2(i) and 2(j)). When sh-circHUWE1 was transfected in, migration and invasion inhibition were observed in A549/DDP and H1299/DDP cells (Figures 2(k)–2(n)). The protein levels of PCNA and MMP 13 were decreased while c-caspase 3 was increased in circHUWE1-silencing A549/DDP and H1299/DDP cells (Figures 2(o)–2(q)). Therefore, knockdown of circHUWE1 has the potency to suppress the progression of DDP-resistant NSCLC.

### 3.3. circHUWE1 Regulated miR-34a-5p Expression in NSCLC Cells

**Figure 3:**
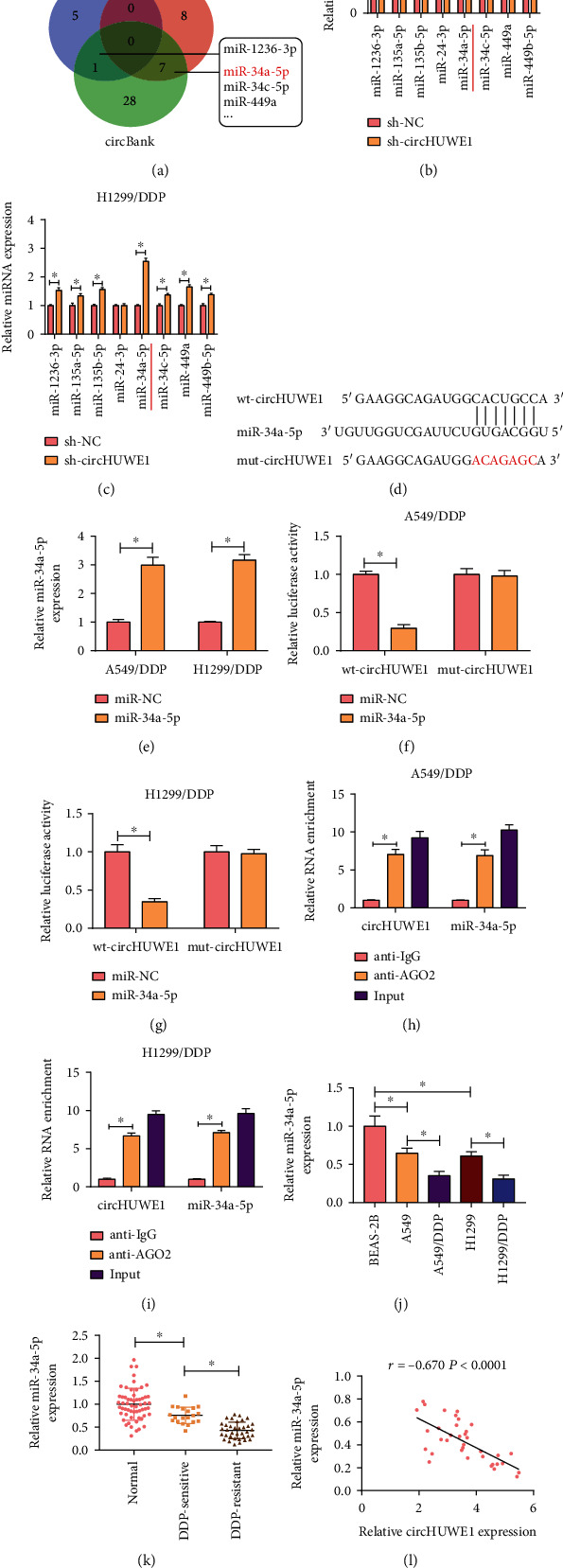
miR-34a-5p was a target of circHUWE1. (a) The candidate miRNAs were enriched by Circinteractome, Starbase, and circBank. (b, c) The expression levels of candidate miRNAs were assessed by RT-qPCR in 549/DDP and H1299/DDP cells transfected with sh-circHUWE1 or sh-NC. (d) miR-34a-5p binding sites on circHUWE1 were presented. (e) miR-34a-5p level was examined by RT-qPCR in 549/DDP and H1299/DDP cells transfected with miR-34a-5p or miR-NC. (f–i) Dual-luciferase reporter assay and RIP assays were performed in 549/DDP and H1299/DDP cells. (j, k) The expression level of miR-34a-5p was examined by RT-qPCR in NSCLC tissues and cells. (l) The correlation between miR-34a-5p and circHUWE1 was analyzed by Pearson's correlation analysis. ^∗^*P* < 0.05.

Using Circinteractome, Starbase, and circBank to cluster candidate target miRNAs of circHUWE1, we identified 8 relational miRNAs, including miR-34a-5p (Figure 3(a)). The silencing of circHUWE1 also obviously enhanced miR-34a-5p expression in 549/DDP and H1299/DDP cells (Figures 3(b) and 3(c)). miR-34a-5p-binding sites on the circHUWE1 is displayed in Figure 3(d), and binding relationship was substantiated by dual-luciferase reporter and RIP assays. The overexpression efficiency of miR-34a-5p was confirmed by RT-qPCR, and miR-34a-5p was upregulated in 549/DDP and H1299/DDP cells after transfection with miR-34a-5p (Figure 3(e)). miR-34a-5p overexpression profoundly reduced the luciferase activity of wt-circHUWE1 but not mut-circHUWE1; in addition, circHUWE1 and miR-34a-5p were greatly enriched by anti-AGO2 (Figures 3(f)–3(i)). Importantly, miR-34a-5p expression was downregulated in NSCLC tissues and cells, especially in DDP resistance tissues and cells (Figures 3(j) and 3(k)). In addition, an inverse correlation between circHUWE1 and miR-34a-5p was confirmed by Pearson's correlation analysis (Figure 3(l)). These findings showed that miR-34a-5p was a target of circHUWE1.

### 3.4. circHUWE1-Inhibition Induced Effects on DDP-Resistant NSCLC Cells Were Reversed by miR-34a-5p Knockdown

**Figure 4:**
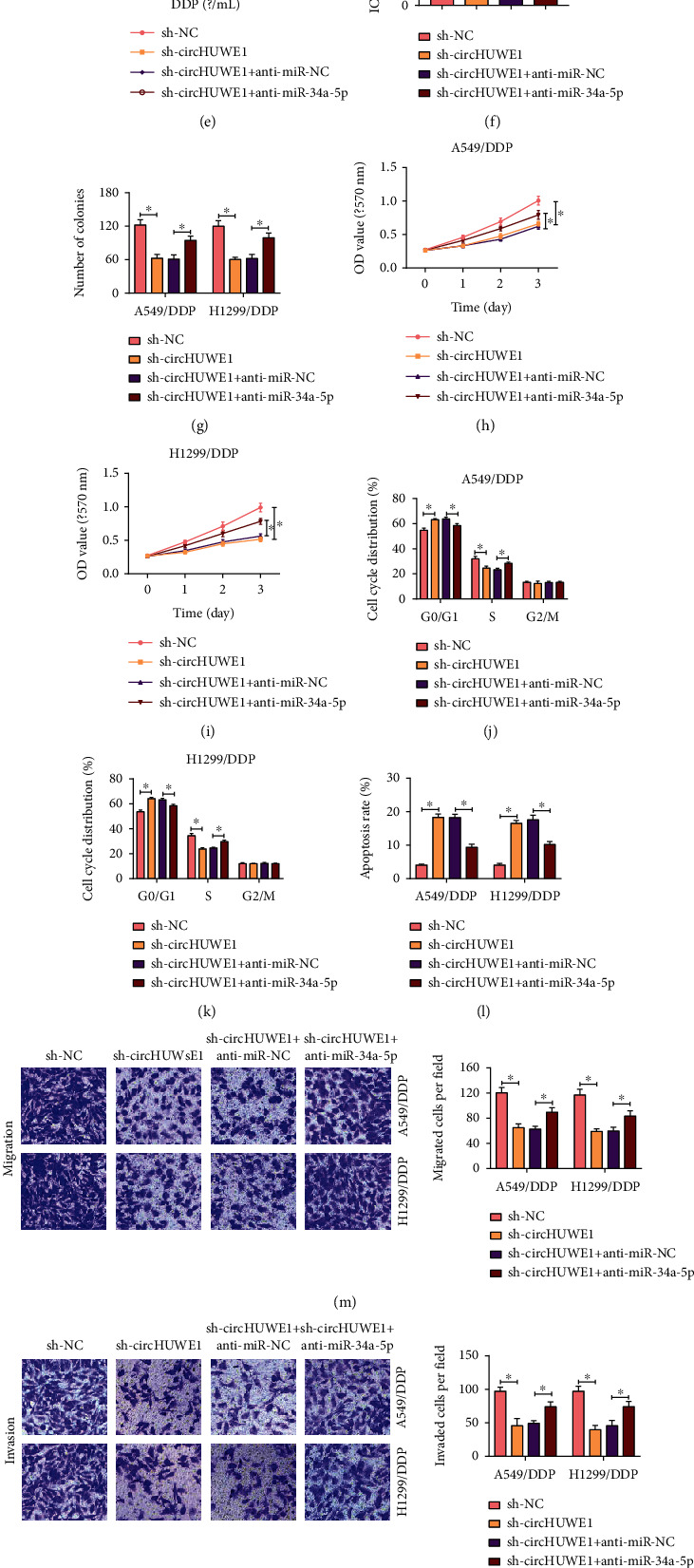
Knockdown of miR-34a-5p abolished circHUWE1-silencing-mediated effects on NSCLC cells. (a) RT-qPCR was employed to detect miR-34a-5p level in A549/DDP and H1299/DDP cells transfected with anti-miR-NC or anti-miR-34a-5p. (b–q) A549/DDP and H1299/DDP cells were transfected with sh-NC, sh-circHUWE1, sh-circHUWE1+anti-miR-NC, or sh-circHUWE1+anti-miR-34a-5p. (b) miR-34a-5p level was assessed by RT-qPCR. (c–f) CCK-8 assay was applied to test IC_50_ of DDP in A549/DDP and H1299/DDP cells. (g–i) The proliferation ability of A549/DDP and H1299/DDP cells was examined by colony forming and CCK-8 assays. (j–l) Flow cytometry was implemented to analyze cell apoptosis and cell cycle distribution. (m, n) Transwell assay was conducted in A549/DDP and H1299/DDP cells. (o–q) Western blot assay was used to assess levels of PCNA, c-caspase 3, and MMP 13. ^∗^*P* < 0.05.

We next explored the biological relevance of circHUWE1 and miR-34a-5p in NSCLC. RT-qPCR assay showed that transfection with anti-miR-34a-5p significantly decreased miR-34a-5p expression in A549/DDP and H1299/DDP cells (Figure 4(a)). Besides, interference of circHUWE1 increased miR-34a-5p level in A549/DDP and H1299/DDP cells, which was weakened by anti-miR-34a-5p (Figure 4(b)). CCK-8 assay revealed that circHUWE1 knockdown attenuated the increase IC_50_ of DDP in A549/DDP and H1299/DDP cells, and this effect was counteracted by anti-miR-34a-5p (Figures 4(c)–4(f)). The cell proliferation inhibition induced by circHUWE1 knockdown in A549/DDP and H1299/DDP was reversed by inhibition of miR-34a-5p (Figures 4(g)–4(i)). We also found cell cycle arrest and increased apoptosis in the sh-circHUWE1-transfected group, which was rescued by cotransfection with anti-miR-34a-5p (Figures 4(j)–4(l)). Transwell assay described that miR-34a-5p inhibition abrogated circHUWE1 deficiency-induced suppressive effects on cell migration and invasion (Figures 4(m) and 4(n)). The downregulation of PCNA and MMP 13 and upregulation of c-caspase 3 were found in circHUWE1-silencing A549/DDP and H1299/DDP cells, which was rescued by miR-34a-5p inhibition (Figures 4(o)–4(q)). Conclusively, knockdown of circHUWE1 suppressed DDP-resistant NSCLC progression through miR-34a-5p.

### 3.5. miR-34a-5p Downregulated TNFAIP8 Expression in NSCLC Cells

**Figure 5:**
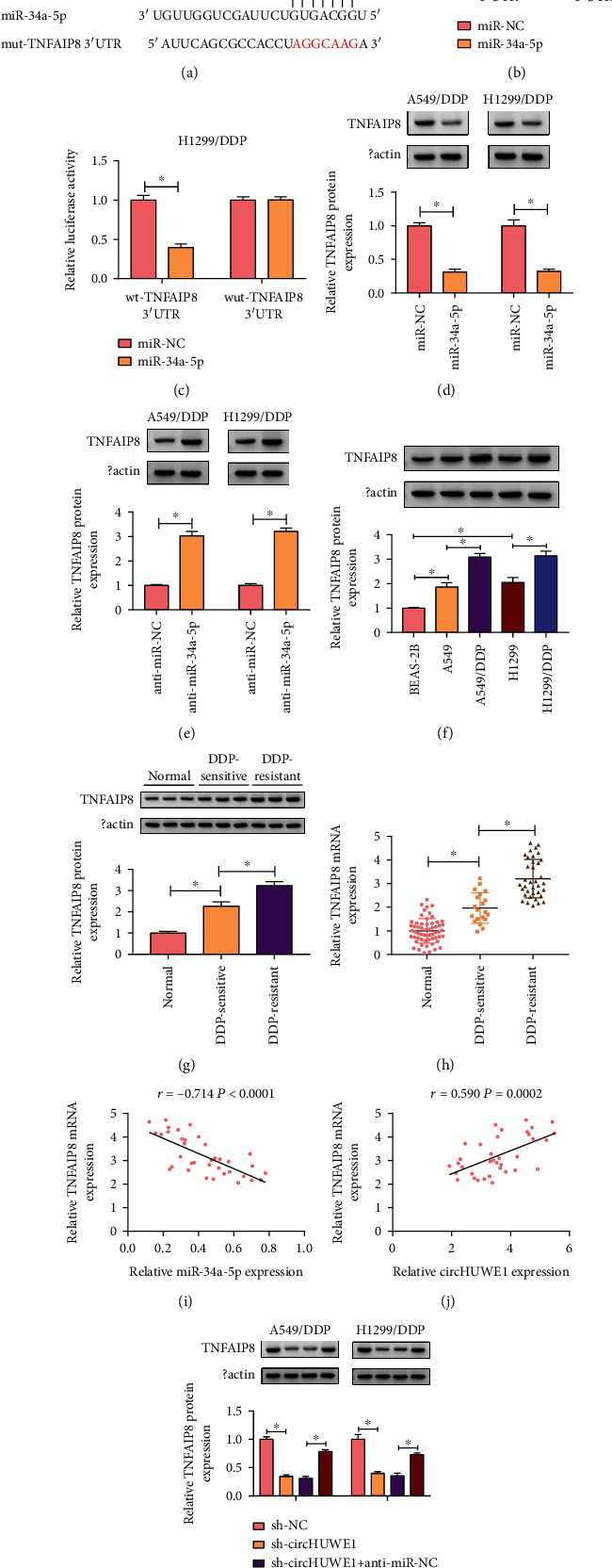
miR-34a-5p targeted TNFAIP8 in NSCLC cells. (a) miR-34a-5p binding sites on 3′UTR of TNFAIP8 were presented. (b, c) Dual-luciferase reporter assay was used to analyze the association between miR-34a-5p and TNFAIP8. (d, e) The expression level of TNFAIP8 was assessed by western blot assay in A549/DDP and H1299/DDP cells transfected with miR-NC, miR-34a-5p, anti-miR-NC, or anti-miR-34a-5p. (f–h) RT-qPCR and western blot assays were used to assess the expression levels of TNFAIP8 in NSCLC tissues and cells. (i, j) Pearson's correlation analysis was used to assess correlation between TNFAIP8 and miR-34a-5p or circHUWE1. (k) The expression level of TNFAIP8 was examined by western blot in A549/DDP and H1299/DDP cells transfected with sh-NC, sh-circHUWE1, sh-circHUWE1+anti-miR-NC, or sh-circHUWE1+anti-miR-34a-5p. ^∗^*P* < 0.05.

The target of miR-34a-5p was assessed via Starbase prediction tool, and binding sites between TNFAIP8 and miR-34a-5p are displayed in Figure 5(a). Furthermore, significant decrease in luciferase activity was found in 549/DDP and H1299/DDP cells cotransfected with miR-34a-5p and wt-TNFAIP8 3′UTR plasmid compared with control (Figures 5(b) and 5(c)). After transfection with miR-34a-5p mimic, the expression of TNFAIP8 was conspicuously reduced in 549/DDP and H1299/DDP cells, while TNFAIP8 was increased in anti-miR-34a-5p-transfected cells (Figures 5(d) and 5(e)). The mRNA and protein levels of TNFAIP8 were increased in NSCLC tissues and cells, and the DDP resistance groups showed the highest expression of TNFAIP8 in all test groups (Figures 5(f)–5(h)). TNFAIP8 was negatively correlated with miR-34a-5p while it was positively correlated with circHUWE1 expression (Figures 5(i) and 5(j)). Importantly, the downregulation of TNFAIP8 in sh-circHUWE1-transfected cells was abolished by an miR-34a-5p inhibitor (Figure 5(k)). TNFAIP8 functioned as a target of miR-34a-5p in NSCLC cells.

### 3.6. miR-34a-5p Could Bind to TNFAIP8 and Acted as a Tumor Suppresser in DDP-Resistant NSCLC Cells

**Figure 6:**
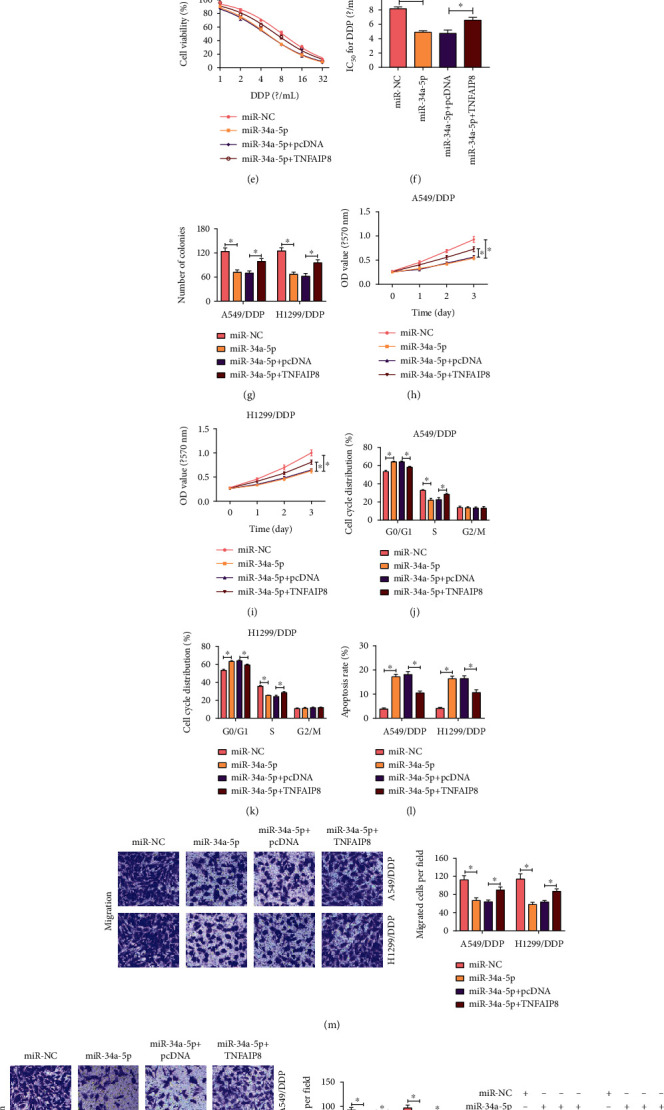
The upregulation of miR-34a-5p-induced effects on NSCLC cells were abolished by TNFAIP8 overexpression. (a) The overexpression efficiency of TNFAIP8 was checked by RT-qPCR. (b–q) A549/DDP and H1299/DDP cells were transfected with miR-NC, miR-34a-5p, miR-34a-5p+pcDNA, or miR-34a-5p+TNFAIP8. (b) Western blot was conducted to assess TNFAIP8 level in A549/DDP and H1299/DDP cells. (c–i) IC_50_ of DDP and proliferation of A549/DDP and H1299/DDP cells were assessed by colony forming and CCK-8 assays. (j–l) Cell apoptosis and cell cycle distribution were evaluated by flow cytometry. (m, n) Transwell assay applied to measure migration and invasion of A549/DDP and H1299/DDP cells. (o–q) The levels of PCNA, c-caspase 3, and MMP 13 were quantified by western blot assay in A549/DDP and H1299/DDP cells. ^∗^*P* < 0.05.

To understand the mechanism by which miR-34a-5p suppressed NSCLC progression, we hypothesized that TNFAIP8 was a target of miR-34a-5p. The association between TNFAIP8 and miR-34a-5p was explored. TNFAIP8 was upregulated in A549/DDP and H1299/DDP cells after transfection with TNFAIP8 (Figure 6(a)). Transfection with TNFAIP8 into 549/DDP and H1299/DDP cells could rescue the downregulation on of TNFAIP8 induced by miR-34a-5p (Figure 6(b)). IC_50_ of DDP in A549/DDP and H1299/DDP cells was reduced by miR-34a-5p mimic but elevated by cotransfecting with TNFAIP8 (Figures 6(c)–6(f)). Not surprising, cell proliferation was inhibited by miR-34a-5p overexpression and rescued by transfecting with TNFAIP8 into A549/DDP and H1299/DDP cells (Figures 6(g)–6(i)). When transfecting with miR-34a-5p mimic, cell cycle was arrested while apoptosis was enhanced in A549/DDP and H1299/DDP cells, which was abrogated by overexpression of TNFAIP8 (Figures 6(j)–6(l)). Migration and invasion were inhibited in miR-34a-5p-induced A549/DDP and H1299/DDP cells while TNFAIP8 upregulation effectively reversed the suppressive effects on migration and invasion (Figures 6(m) and 6(n)). The miR-34a-5p upregulation decreased PCNA and MMP 13 while increased c-caspase 3 in A549/DDP and H1299/DDP cells, which was overturned by TNFAIP8 upregulation (Figures 6(o)–6(q)). These results suggested that miR-34a-5p impeded DDP-resistant NSCLC progression by decreasing TNFAIP8 expression.

### 3.7. Silencing of circHUWE1 Repressed DDP-Resistant NSCLC Cell Proliferation *In Vivo*

**Figure 7:**
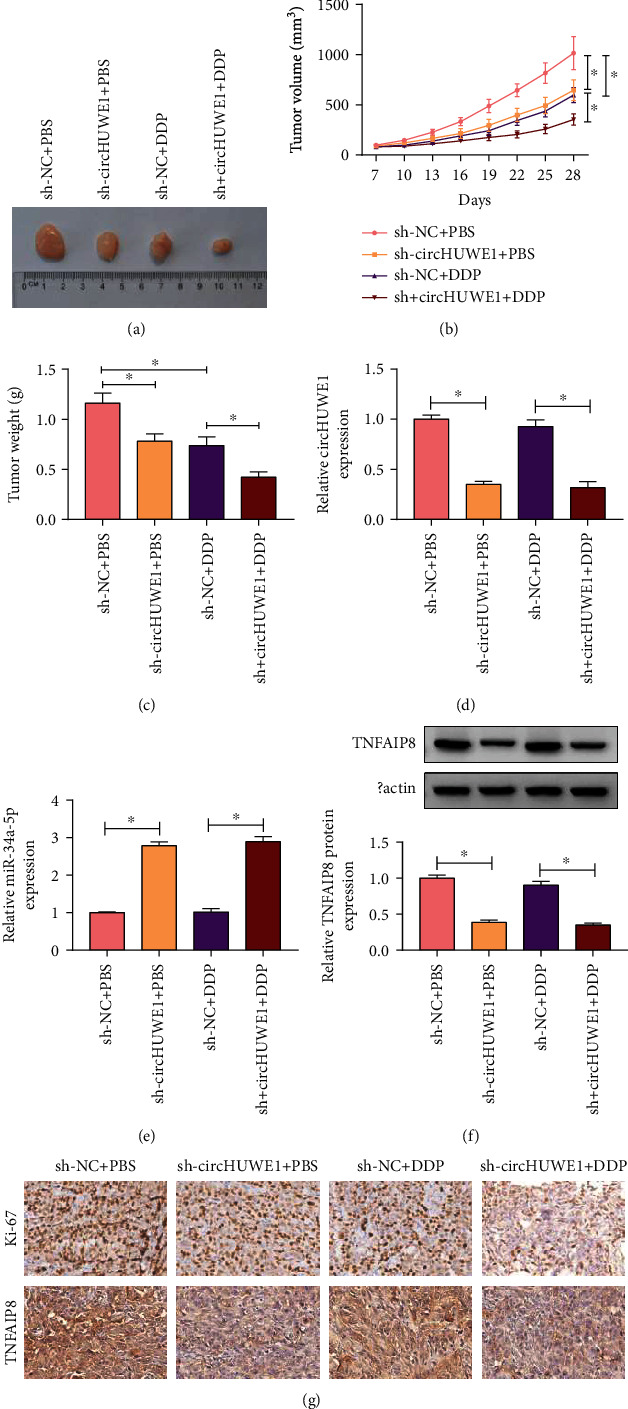
Silencing of circHUWE1 repressed xenograft growth. (a–c) Representative images of removed tumor tissues, tumor volume, and weight were presented. (d–f) The expression levels of circHUWE1, miR-34a-5p, and TNFAIP8 were assessed by RT-qPCR and western blot. (g) The immunochemistry for Ki-67 and TNFAIP8 was performed. ^∗^*P* < 0.05.

A xenograft model was performed to analyze the effects of circHUWE1 silencing on xenograft growth. As presented in Figures 7(a)–7(c), tumor growth and tumor weight were significantly inhibited in the sh-circHUWE1 group compared with the sh-NC group, and the inhibitory effects of circHUWE1 silencing on tumor growth were enhanced by treatment with DDP treatment. Besides, the expression of circHUWE1 and TNFAIP8 was decreased whereas that of miR-34a-5p was increased in removed tissues collected from the sh-circHUWE1 and sh-circHUWE1+DDP groups compared with matched controls (Figures 7(d)–7(f)). In addition, the immunochemistry suggested a significant decrease in both Ki-67 and TNFAIP8 expression in the circHUWE1 knockdown group compared to controls (Figure 7(g)). Taken together, circHUWE1 silencing suppressed tumor growth of DDP-resistant NSCLC *in vivo*.

## 4. Discussion

circRNAs had been declared to participate in drug resistance in NSCLC. For example, NSCLC cell-derived exosomes containing high circ-CPA4 level induced stemness and increased the innate DDP resistance in both A549 and H1299 cells through the let-7 miRNA/PD-L1 axis [27]. circSMARCA5 was originally identified in glioblastoma multiforme [28], and its overexpression was inclined to increase sensitivity to cisplatin in breast cancer MCF7 cells by interacting with host gene [29]. In terms of our research, circHUWE1 was found to be highly expressed in NSCLC and was related to DDP resistance. Function analysis experiments demonstrated that circHUWE1 downregulation inhibited DDP resistance and malignant phenotypes of DDP resistance-acquired NSCLC A549 and H1299 cells, which was dependent on regulating miR-34a-5p and TNFAIP8.

DDP treatment is a common way for tumor chemotherapy, such as lung cancer, but successful chemotherapy is still not satisfied following the development of multidrug resistance [30, 31]. Thus, exploring the pathogenesis mechanism and DDP resistance was important to identify possible therapeutic strategy for NSCLC. According to a previous report, circHUWE1 was regarded as an oncogene in regulating the invasion and proliferation of colorectal cancer cells [32]. By the way, little literatures had been reported to describe a circHUWE1 role in human cancers, except for colorectal cancer [32] and NSCLC (this study). Mechanically, the putative function of circRNA includes serving as miRNA sponges, and the cross-talk between circRNA and miRNAs is of great importance in the progression of tumorigenesis [33]; however, whether circHUWE1 functioned in NSCLC through sponging miRNAs remained unclear before this study. Here, we identified circHUWE1 as a key regulator of the progression of DDP-resistant NSCLC by sponging miR-34a-5p.

miR-34a-5p belongs to the miR-34 miRNA family, and most of them promote tumor development by exerting an antioncogenic role in tumorigenesis [34–36]. Supporting a tumor-inhibitory role for miR-34a-5p in NSCLC, luteolin exerted significant cancer inhibitory activity through upregulation of miR-34a-5p level in NSCLC [37]. Importantly, the antitumor roles of miR-34a-5p were implicated in DDP resistance [38, 39]. Consistent with previous conclusions, we verified that miR-34a-5p acted as an inhibitor for DDP resistance in NSCLC by negatively regulating the expression of TNFAIP8.

Together, these findings identified a previously unknown regulatory axis circHUWE1/miR-34a-5p/TNFAIP8 in DDP-resistant NSCLC progression, representing the first functional characterization of circHUWE1 and confirming it as a key driver for DDP resistance.

By the way, emerging role of circRNAs [40], long noncoding RNAs [41, 42], and miRNAs [43] had been well-documented in cancer development and progression. Moreover, serum extracellular vesicle- (EV-) derived circRNAs could be highly stable minimally invasive diagnostic biomarkers [44]. Nevertheless, this study did not further explore a circHUWE1 role in NSCLC A549 and H1299 cells and circHUWE1 expression in EV from patients' blood and cell culture medium, at least temporarily.

## 5. Conclusion

In summary, circHUWE1 downregulation inhibited DDP resistance, proliferation, migration, and invasion while it induced apoptosis and cell cycle arrest of DDP resistance-acquired NSCLC cells depending on the regulation of the miR-34a-5p/TNFAIP8 axis, supporting an important role of circHUWE1 in DDP-resistant NSCLC progression. This work provided a novel promising target for DDP-resistant NSCLC diagnosis.

## Data Availability

No data were used to support this study.
